# Anti-Tumor Drug Discovery Based on Natural Product β-Elemene: Anti-Tumor Mechanisms and Structural Modification

**DOI:** 10.3390/molecules26061499

**Published:** 2021-03-10

**Authors:** Ziqiang Bai, Chuansheng Yao, Junlong Zhu, Yuanyuan Xie, Xiang-Yang Ye, Renren Bai, Tian Xie

**Affiliations:** 1 College of Pharmaceutical Science, Zhejiang University of Technology, Hangzhou 310014, China; bzq202020@163.com (Z.B.); yaochuansheng95@126.com (C.Y.); xyycz@zjut.edu.cn (Y.X.); 2 College of Pharmacy, School of Medicine, Hangzhou Normal University, Hangzhou 311121, China; zjl790389369@163.com; 3 Key Laboratory of Elemene Class Anti-Cancer Chinese Medicines; Engineering Laboratory of Development and Application of Traditional Chinese Medicines; Collaborative Innovation Center of Traditional Chinese Medicines of Zhejiang Province, Hangzhou Normal University, Hangzhou 311121, China

**Keywords:** natural product, β-elemene, anti-tumor, mechanism, structural modification

## Abstract

Natural products are important sources for drug discovery, especially anti-tumor drugs. β-Elemene, the prominent active ingredient extract from the rhizome of *Curcuma wenyujin*, is a representative natural product with broad anti-tumor activities. The main molecular mechanism of β-elemene is to inhibit tumor growth and proliferation, induce apoptosis, inhibit tumor cell invasion and metastasis, enhance the sensitivity of chemoradiotherapy, regulate the immune system, and reverse multidrug resistance (MDR). Elemene oral emulsion and elemene injection were approved by the China Food and Drug Administration (CFDA) for the treatment of various cancers and bone metastasis in 1994. However, the lipophilicity and low bioavailability limit its application. To discover better β-elemene-derived anti-tumor drugs with satisfying drug-like properties, researchers have modified its structure under the premise of not damaging the basic scaffold structure. In this review, we comprehensively discuss and summarize the potential anti-tumor mechanisms and the progress of structural modifications of β-elemene.

## 1. Introduction

Cancer generally refers to various malignant tumors and is characterized by deregulated cellular behavior [1]. There were about 14.1 million new cancer cases and 8.2 million deaths worldwide in 2012 based on GLOBOCAN data [2]. It was estimated that the numbers of new cancer cases and cancer deaths were 4.3 and 2.8 million in China in 2015, respectively [3]. Surgery, radiotherapy, and chemotherapy are currently the main treatments for cancer, which can effectively kill cancer cells but also cause serious adverse reactions due to the intervention affecting normal cells. Another problem with cancer therapy is the development of drug resistance during the treatment course. Therefore, the development of more effective treatment with fewer side effects and retarded resistance development is an urgent need. Chinese herbal medicine has been used to treat many diseases, including cancer, for thousands of years, and its effective ingredients have always been the research focus of researchers [4].

**Figure 1:**
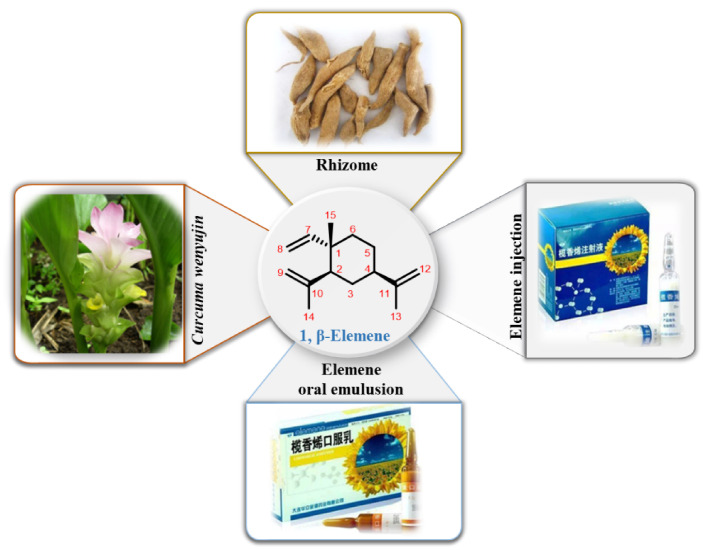
The plant source, commercial products, and the structure of β-elemene.

Our research group has been focused on the investigation of the anti-tumor activity of the Chinese herbal medicine *Curcuma wenyujin*. After efforts of many years, we successfully separated and identified the anti-tumor natural product β-elemene (1-methyl-1-vinyl-2,4-diisopropenyl-cyclohexane, C_15_H_24_, MW: 204.35, Figure 1), the prominent active ingredient extract from the rhizome of *Curcuma wenyujin*. Numerous experiments were subsequently performed to prove its promising in vitro and in vivo anti-tumor effects, leading to the approval of elemene oral emulsion (CFDA number H20010338) and elemene injection (CFDA number H10960114) by the China Food and Drug Administration (CFDA) as a broad spectrum of the anti-tumor drugs for the treatment of lung cancer, liver cancer, esophageal cancer, nasopharyngeal cancer, brain cancer, and bone metastasis in 1994. These two common dosage forms have been currently applied in clinical for more than 20 years [5,6]. Their specific information is summarized in Table 1. 

Compared with radiotherapy and chemotherapy, elemene treatment displayed fewer side effects and is tolerated by most patients [7]. However, the lipophilicity and low bioavailability limit its application. As a sesquiterpenoid volatile oil, its chemical structure contains only two elements of hydrocarbon and hydrogen, resulting in poor aqueous solubility. Therefore, β-elemene is a lead compound to discover better anti-tumor drugs with satisfying drug-like properties. Because the three olefinic bonds play important roles in its anti-tumor activity, the researchers have modified its structure under the premise of retaining the basic scaffold structure and the double bonds, including introducing hydrophilic moieties like hydroxyl and amino groups to improve its water solubility.

In this review, we discuss and summarize the potential anti-tumor mechanisms and the progress of structural modifications of β-elemene.

## 2. The Potential Anti-Tumor Mechanisms of β-Elemene

**Figure 2:**
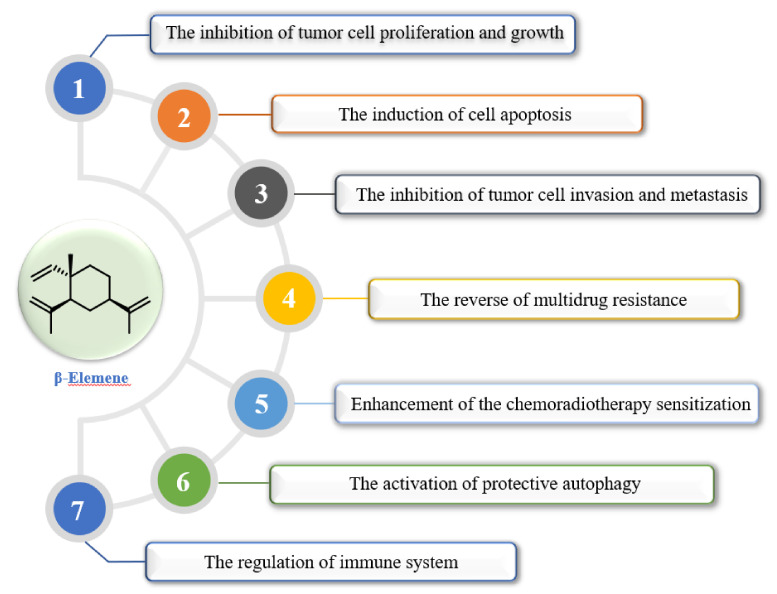
Summary of the potential anti-tumor mechanisms of β-elemene.

β-Elemene shows various anti-tumor effects in different tumor cells and the exact anti-tumor mechanisms have not been fully elucidated. The main molecular mechanism is to inhibit tumor growth and proliferation, induce apoptosis, and inhibit tumor cell invasion and metastasis. β-Elemene involves the regulation of various signal pathways and enzymes/proteins including p38-MAPK, Wnt-β-catenin, PI3K-AKT-Mammalian target of rapamycin (mTOR), and Bcl-2 protein family and caspase. Moreover, β-elemene can also enhance the sensitivity of chemotherapy or radiotherapy, regulate the immune system, and reverse multidrug resistance (MDR). The potential anti-tumor mechanisms of β-elemene are summarized in Figure 2.

### 2.1. The Inhibition of Tumor Cell Proliferation and Growth

A cell cycle is mainly divided into the G_0_ phase (quiescent stage), G_1_ phase (the early stage of DNA synthesis), S phase (DNA synthesis period), G_2_ phase (the later stage of DNA synthesis), and M phase (the mitotic stage). Cyclin-dependent kinases (CDK) are a group of serine/threonine protein kinases, which can advance and transform the cell cycle in different phases by cooperating with cyclin. Cell division cycle (CDC) genes and CDK inhibitors (p21, p27, p15, and p16) play an important role in the regulation of the cell cycle [8]. Tumor cells grow vigorously and lose control with relative autonomy. β-Elemene was reported to inhibit the proliferation of non-small-cell lung cancer (NSCLC) cell line H460 and concentration-dependently induce cell cycle arrest at the G_2_/M phase. Further mechanism studies indicated that β-elemene could reduce the activities of cyclin-CDC/CDK complexes including cyclin B1-CDC2, cyclin A-CDC2, and cyclin A-CDK2, leading to arrest cell cycle at the G_2_/M phase [9].

Topoisomerase I (TOPO I) and TOPO IIα are key enzymes that regulate the topological configuration of nucleic acids and have become important targets for tumor chemotherapy [10]. In human hepatoma HepG2 cells, β-elemene was found to concentration- and time-dependently inhibit cell proliferation and arrest the cell cycle at the G_2_-M/S phase. These effects were mediated by the downregulation of the protein expression of TOPO I and TOPO IIα [11,12]. It was also reported that β-elemene could induce the inhibition of murine hepatocellular carcinoma cell line H22 by downregulating the expression of c-Met and enhancing the protein expression of histone H1 [13,14]. In human glioblastoma C6 and U251 cells, β-elemene was found to arrest the cell cycle at the G_0_/G_1_ phase and inhibit cell proliferation through phosphorylation of the p38 MAPK pathway, which was blocked by SB203580, an inhibitor of p38 MAPK kinase [15]. In addition, β-elemene could also decrease cell viability and tumor volume via activating the glia maturation factor β (GMFβ)-dependent MKK3/6-p38 signaling pathway activation as well as inhibiting the ERK1/2-Bcl-2/survivin signaling pathway in glioblastoma U87 cells [16,17]. 

Telomerase is an enzyme responsible for telomere lengthening in cells and involves maintaining chromosomal stability and cell viability. The expression of telomerase is markedly upregulated in many tumor cells, resulting in unrestricted growth and proliferation of these cells. Human telomerase reverse transcriptase (hTERT) expression shows a positive correlation with telomerase activity [18]. β-Elemene was found to suppress esophageal carcinoma ECA-109 cell proliferation by inhibiting the expression of hTERT mediated by long noncoding RNA (lncRNA) CDKN2B-AS1 [19]. The epigenetic inactivation of cancer-related genes is closely related to DNA hypermethylation and these abnormal epigenetic modifications are associated with DNA methyltransferase (DNMT) [20]. β-Elemene could also inhibit the growth of C666-1 and HNE2 cells (nasopharyngeal carcinoma cells) associated with the inactivation of signal transducer and activator of transcription 3 (Stat3) and the reduced expression of DNMT1 and enhancer of zeste homolog 2 (EZH2) [21]. In human NSCLC A459 and PC9 cells, β-elemene inhibited cell growth via reducing the protein expression of DNMT1 mediated by ERK1/2 and AMPKα signaling pathways [22]. Subsequent investigations demonstrated that β-elemene increased insulin-like growth factor-binding proteins 1 (IGFBP 1) gene expression through inactivating Stat3 as well as the interaction between miRNA155-5p and human forkhead box class O 3a (FoxO 3a), leading to the inhibition of human lung cancer A549 and H1975 cell growth [23]. Another study indicated that β-elemene selectively inhibited the proliferation of glioma stem-like cells (GSLCs), including U87, U373, SHG-44, T98G, and SKMG-4 cells compared with parental glioma cells associated with downregulating Notch1 expression [24]. Moreover, β-elemene also inhibited bladder carcinoma T24 cell proliferation and induced apoptosis through upregulating Smad4 gene expression, a tumor suppressor gene [25]. 

Wnt-Frizzled-β-catenin signaling transduction pathway plays a significant role in proliferation, differentiation, and orientation by regulating its downstream target molecule, including TCF7, c-Myc, and cyclin D1 [26]. A study indicated that administration of β-elemene concentration- and time-dependently inhibits human cervical cancer SiHa cell proliferation and arrests the cell cycle at the G_1_ phase through the upregulation of P15 expression and the downregulation of cyclin D1 expression. Moreover, β-elemene also inhibits cell invasion and apoptosis via the Wnt-Frizzled-β-catenin signaling transduction pathway [27]. 

The microtubule is an important part of the cytoskeleton. Interferences with the polymerization and decomposition of microtubules are a vital target for tumor drug therapy. β-Elemene injection effectively inhibits hepatoma HepG2 cell proliferation and arrests the cell cycle at the S phase associated with downregulating alpha-tubulin and inhibiting microtubular polymerization [28].

### 2.2. The Induction of Cell Apoptosis

Apoptosis is a genes-controlled autonomous and orderly death process of cells, playing a pivotal role in cancer regulation [29]. The death receptor pathway (for example, Fas/APO1 and APO3), the mitochondrial pathway, and the endoplasmic reticulum (ER) stress pathway are the main apoptosis pathways. Cysteinyl aspartate-specific proteinase (caspase) plays a critical regulatory role in all apoptosis pathways. In the FasL/Fas signaling pathway, the fas-associated death domain (FADD) leads to the cleavage of procaspase-8 and activation of caspase-8, which then activates downstream caspases (caspase-3 and caspase-7) and causes cell apoptosis. On the other hand, activated caspase-8 will cleave binding interface database (Bid) into truncated binding interface database (tBid), which in turn induces the release of mitochondrial cytochrome C into the cytoplasm, and the apoptosis through the mitochondrial pathway [30]. In addition, DNA damage and induction of chemotherapeutic drugs and radiation can cause a decrease in mitochondrial transmembrane potential and an increase in membrane permeability, leading to the release of cytochrome C into the cytoplasm. The released cytochrome C then binds to apoptosis protease-activating factor-1 (Apaf-1) to recruit procaspase-9, forming an apoptosome, which causes the self-clearage and self-activation of procaspase-9. Activated caspase-9 initiates the downstream caspase cascade to induce apoptosis. The X-linked inhibitor of apoptosis (XIAP) and the Bcl-2 family proteins, including Bcl-2, Bcl-XL, Bax, and Bad, tightly participate in the regulation of the apoptosis process [31,32]. 

A study indicated that β-elemene could effectively inhibit NSCLC A549 cells’ vitality with an IC_50_ value of 27.5 μg/mL. The effect was associated with ER stress activation via the PERK/IRE1α/ATF6 pathway mediated by reactive oxygen species (ROS) generation. The anti-tumor effect of β-elemene was also confirmed in Lewis tumor-bearing C57BL/6J mice [33]. A subsequent study demonstrated that Cb1-regulated AKT and ERK signaling pathways are associated with β-elemene-induced apoptosis. Moreover, the decreased Bcl-2 expression, increased Bax expression, and the cleavage of poly ADP-ribose polymerase (PARP) also contribute to β-elemene-induced apoptosis in A549 cells [34]. β-Elemene also induces gastric cancer SGC7901 cell apoptosis in a concentration-dependent manner. 147 Upregulated proteins and 86 downregulated proteins as well as several pathways, including ribosome signaling, peroxisome proliferator-activated receptors (PPARs) signaling pathway, and p21-activated protein kinase 1 (PAK1) were responsive to the treatment of β-elemene [35]. 

Survivin, abundantly expressed in tumors, is a member of the inhibitor of apoptosis (IAP) gene family [36]. In human glioma U251 and A172 cells, β-elemene induced cell apoptosis associated with the expression inhibition of the survivin gene and the interaction between survivin and hepatitis B X-interacting protein (HBXIP). The apoptotic effect was also associated with the activation of caspase-3/-7/-9 and increased levels of cleaved PARP [37]. In addition, treatment with β-elemene induced human glioblastoma multiform cell line U87MG by disrupting the formation of the Hsp90/Raf-1 complex, leading to Raf-1 deactivation and ERK pathway inhibition [38]. In bladder cancer T24 cells, β-elemene could induce apoptosis through downregulating the expression of several substances, including survivin, Bcl-XL, and metastasis-associated gene 1 (Mta-1). The anti-tumor effect of β-elemene was dependent on the dosage and length of incubation time [39]. 

The BH3-only protein p53-up-regulated modulator of apoptosis (PUMA) is a downstream effector in the transforming growth factor-β (TGF-β)-induced apoptosis pathway in myc-driven B-cell lymphomas [40]. β-Elemene significantly causes the apoptosis of Burkitt’s lymphoma Raji in a concentration-dependent manner via increasing the expression of PUMA, Bax, Bak, Bim, and Bid, as well as decreasing Bcl-2 and Bcl-XL expression [41]. 

### 2.3. The Inhibition of Tumor Cell Invasion and Metastasis

The metastasis of tumor cells is the end product of the invasion-metastasis cascade, including the formation of the primary tumor, local invasion, intravasation, survival in the circulation, extravasation, and metastasis. Several substances are involved in the cascade process. Among them, matrix metalloproteinases (MMPs) destroy the tissue barrier of tumor cell invasion and epithelial-mesenchymal transition (EMT) endows cells the ability of metastasis and invasion. Moreover, angiogenesis and angiogenesis-related factors include vascular endothelial growth factor (VEGF) and basic fibroblast growth factor (bFGF), laying a foundation for metastasis [42].

Researchers have shown that β-elemene can effectively inhibit tumor cell invasion and metastasis in a variety of ways. It has been reported that β-elemene could inhibit melanoma B16F10 cell metastasis via downregulating the mRNA and protein expression of MMP-2/9, urokinase-type plasminogen activator (uPA), and its receptor (uPAR) both in vitro and in vivo [43,44]. β-Elemene can also inhibit the growth of and metastasis of melanoma B16F10 cells through suppressing VEGF-mediated angiogenesis and the expression of CD34, a key marker of primary melanoma angiogenesis [45]. In CD44+ gastric cancer stem-like cells (GCSCs), β-elemene effectively attenuated cell angiogenesis by interfering Notch-1 expression but not with DII4 [46]. In gastric cancer cell lines, BGC823 and SGC7901, β-elemene was found to inhibit peritoneal metastasis by modulating the focal adhesion kinase (FAK)/Claudin-1 signaling pathway, including downregulated FAK phosphorylation and Claudin-1 expression [47]. Moreover, β-elemene effectively suppresses cell metastasis through inhibiting the expression of miR-1323 and then upregulating Cbl-b expression, leading to the blocking of the EGFR-ERK/AKT signaling pathways in the doxorubicin (Adriamycin)-resistant variant of SGC7901. Taken together, its anti-metastasis activity of MDR gastric cancer cells was associated with the miR-1323/Cbl-b/EGFR signaling axis [48]. 

E-cadherin is a member of the cadherin family and its inactivation results in cell migration and invasion and is involved in the metastasis of cancer cells. β-Elemene could decrease cell migration and invasion by upregulating the mRNA and protein expression of E-cadherin via upregulating estrogen receptor-α and metastasis-associated protein 3 (MAT3) and decreasing the nuclear transcription factor Snail in human breast cancer cell line MCF-7 [49]. Further investigations showed that β-elemene also blocked EMT through decreasing Smad3 expression and phosphorylation, leading to the inhibition of TGF-β1-mediated upregulation of mRNA and protein expression of nuclear transcription factors, including Snail/SNAI1, Slug/SNAI2, TWIST, and SIP1 [50]. Furthermore, β-elemene was found to inhibit human breast cancer MDA-MB-231 and MCF-7 cells metastasis by blocking dimeric pyruvate kinase M2 (PKM2) transformation and nuclear translocation mediated aerobic glycolysis [51].

### 2.4. The Reverse of Multidrug Resistance

Multidrug resistance, the simultaneous development of resistance to different drugs with different targets and chemical structures, is one of the important reasons for the failure of tumor chemotherapy. The increased efflux of various hydrophobic cytotoxic drugs mediated by the ATP-binding cassette (ABC) transporters, including P-glycoprotein (P-gp, also known as MDR1 or ABCB1) and breast cancer resistance protein (BCRP), is a common drug resistance mechanism [52]. Current studies have shown that β-elemene can effectively reverse MDR through inhibiting ABC transporters’ expression and improving the anti-tumor effect.

In cisplatin (DDP)-resistant human lung adenocarcinoma A549/DDP cells, β-elemene could effectively reverse drug resistance by decreasing mitochondrial membrane potential and the expression of the P-gp activated intracellular redox system and activating apoptosis [53]. In drug-resistant leukemia and gastric adenocarcinoma cell lines K562/DNR and SGC7901/ADR, β-elemene significantly enhanced the efficacy of doxorubicin (DOX) efficiency through upregulating the E3 ubiquitin ligases (c-Cb1 and Cbl-b) and then inhibiting the PI3K/AKT signaling pathway, as well as downregulating the expression of P-gp [54]. Moreover, β-elemene also increased the cytotoxicity of multiple chemotherapy drugs (paclitaxel, colchicine, and vinblastine) in overexpressed ABCB1 transporter cell lines (KB-C2, HEK293, and NCI-H460/MX20) associated with inhibiting the activity of ABCB1 transporter [55]. It was also demonstrated that treatment with β-elemene could inhibit the proliferation of the cisplatin-sensitive/resistant human ovarian cancer cell line A2780 and A2780/CP by downregulating the expression of CDC2 and cyclin A/B1 as well as upregulating p21^WAF1/CIP1^ and p53 protein expression, leading to cell cycle arrest at G_2_/M phase. Further studies indicated that β-elemene also induced apoptosis through activating caspase-3, -8, and -9 [56,57]. These findings showed that β-elemene was a potential agent for the treatment of cisplatin-resistant ovarian cancer.

It has been reported that β-elemene could reverse chemoresistant human breast cancer cells (MCF-7/doxorubicin, MCF-7/adriacin, and MCF-7/docetaxel) associated with the inhibition of BCRP and P-gp transporter expression as well as the increased expression of phosphatase and tensin homolog deleted on chromosome ten (PTEN) [58,59,60,61]. Moreover, β-elemene was also found to reverse drug resistance through the mitochondrial apoptosis pathway in the cisplatin-resistant human lung adenocarcinoma A549/DDP cells and through inducing pro-death autophagy and arresting the cell cycle dependent on cyclin D3 in the 5-fluorouracil resistant human p53-deficient colorectal cancer lines HCT116p53^-/-^ [62,63].

### 2.5. Enhancement of the Chemoradiotherapy Sensitization

Many reports proved that β-elemene could enhance the therapeutic effects of radiotherapy and chemotherapy in different aspects. β-Elemene was found to improve the radiosensitivity (X-ray, 3.38 Gray/min, 2/4/6/10 Gray) and chemosensitivity (temozolomide) of the human glioblastoma multiforme (GBM) cell lines (U87MG, T98, U251, and LN229) associated with the inhibition of DNA damage repair through ATM, AKT, and ERK signaling pathways [64]. β-Elemene also enhanced the efficacy of gefitinib, an epidermal growth factor receptor (EGFR) inhibitor, against GBM U87MG and U251 cells via inhibiting the EGFR signaling pathway [65]. In the human cisplatin-resistant ovarian cancer cell lines A2780/CP70 and MCAS, β-elemene was found to increase the susceptibility to cisplatin through the regulation of DNA repair activity including the expression inhibition of excision repair cross-complementation group-1 (ERCC-1) and the induction of apoptosis, including the increased phosphorylate level of JNK and the downregulation of XIAP expression [66,67]. A study indicated that β-elemene could sensitize the human lung adenocarcinoma A549 cells to radiation (γ-ray, 6 Gray) by upregulating p53 expression, downregulating Bcl-2 expression, and inducing apoptosis [68]. Zou and co-workers have reported that β-elemene displayed a radiosensitization (6 MV, X-ray, 4 Gray) effect in human lung adenocarcinoma A549 cells through facilitating DNA damage and suppressing DNA repair in vitro. These effects may be mediated by inhibiting the mRNA expression of hypoxia inducible factor-1α (HIF-1α), survivin, and mTOR [69,70]. Further studies showed that β-elemene (45 mg/kg, i.p.) significantly improved the radiosensitivity (6 MV electron beam from a linear accelerator, 5 Gray) of A549 cells xenograft in vivo by suppressing the HIF-1α-survivin pathway and directly or indirectly downregulating the mRNA and protein expression of radiation-induced peroxiredoxin-1 (Prx-1), a critical molecule in redox regulation of tumor cells [71,72]. Furthermore, β-elemene also increased cisplatin chemosensitivity of the human NXCLC cell lines (H460 and A549) via inducing mitochondria-mediated intrinsic apoptosis and arresting the cell cycle at the G_2_/M phase associated with the CHK2-mediated CDC25C/CDC2/cyclin B1 signaling pathway [73,74].

In human bladder cancer T24 and 5637 cells, β-elemene could arrest the cell cycle at the G_0_/G_1_ phase and enhance cisplatin-induced apoptosis dependent on caspase activity and the ROS-AMPK signaling pathway [75,76]. β-Elemene was also found to display a radiosensitizer effect in human gastric cancer MKN45 and SGC7901 cells. Further investigations have shown that this effect is mediated by upregulating PAK1-interacting protein 1 (PAK1IP1) expression and downregulating the levels of threonine (T423, phosphor-Pak1) and phosphor-ERK1/2, thereby inhibiting the PAK1 signaling pathway [77]. In the triple-negative breast cancer cells, MDA-MB-231 and BT549, the combination of β-elemene and 5-fluorouracil (5-FU) markedly suppressed cell proliferation, migration, and invasion by inhibiting NF-κB, PI3K/PTEN/AKT/mTOR and Ras-Raf-MEK-ERK signaling pathways. These effects were also confirmed with LY294002 (PI3K inhibitor) and U0126 (Ras-Raf-MEK-ERK inhibitor) [78]. It is also reported that β-elemene remarkably enhances the therapeutic effect of cisplatin on proliferative inhibition and apoptosis via blocking the JAK2-STAT3 signaling pathway, including the inhibition of p-STAT3, p-JAK2, and Bcl-2 expression, as well as the upregulation of Bax and caspase-3 expression [79]. In human melanoma A375 cells, co-treatment of β-elemene with radiation (6 MV, X-ray, 2 and 4 Gray) notably decreased the cell viability by 23% at a dose of 2 Gray and 30% at a dose of 4 Gray [80]. In human colorectal carcinoma HCT116 and HT29 cells, β-elemene was found to improve the effect of 5-FU by downregulating the expression of miR-191, which is associated with the inhibition of the Wnt/β-catenin pathway [81]. Moreover, β-elemene also enhanced cisplatin and oxaliplatin sensitivities as well in the androgen-independent prostate carcinoma cell lines (DU145 and PC-3) and human hepatocellular carcinoma cell lines (Hep3B, Huh7, MHCC97H, and MHCCLM3), respectively [82,83]. β-Elemene could also augment the cisplatin-induced apoptosis dependent on the activation of mitochondria and blocking the reduction of copper transporter 1 (CTR1) following oxaliplatin treatment.

### 2.6. The Activation of Protective Autophagy

Autophagy is a process by which autophagosomes engulf cellular proteins and organelles are delivered to lysosomes for degradation. Autophagy exhibits a controversial dual role (promotion or inhibition) in the occurrence and development of cancer and the detailed mechanisms are largely unclear. Several studies indicated that autophagy pharmacological inhibitors can improve the effects of certain anti-tumor agents [84,85]. The lipid-conjugated form of LC3II by the combination of LC3I, phosphatidylethanolamine (PE), autophagy-related gene 4B (Atg4B), and Atg7 commonly serves as an autophagosome marker [86]. β-Elemene was found to have both the effects of inducing apoptosis and activating protective autophagy. 

The activation of the PI3K-PDK1-AKT-TSC1/2-Rheb-mTORC1-p70S6K-S6 signaling pathway leads to an increase in protein synthesis. Meanwhile, mTOR is also a key regulator, and the activation of mTOR triggers autophagy. β-Elemene was proved to effectively inhibit the viability of various tumor cells, including human NSCLC A549 cells, gastric cancer MGC803 and SGC7901 cells, and renal-cell carcinoma 786-0 cells by suppressing the PI3K/AKT/mTOR signaling pathway. At the same time, it also triggered protective autophagy characterized by the increased punctate LC3 dots and LC3II protein level. Treatment with chlorochine or 3-methyladenine (autophagy inhibitors) or knockdown of Beclin 1 with siRNA significantly enhanced the effect of β-elemene [87,88,89]. These effects were also confirmed in the human hepatoma cancer HepG2 cells and breast cancer cell lines (Bcap37 and MBA-MD-231) [90,91]. These findings indicate that β-elemene in combination with autophagy inhibitors is a feasible way for the therapy of cancer.

### 2.7. The Regulation of the Immune System

The reduction of the immune recognition, the enhancement of tumor cells resistant to immune cells, or the immunosuppression of the tumor microenvironment may mediate escaping from immune control [92]. β-Elemene was found to enhance the effect of the immune system.

Tumor necrosis factor-related apoptosis-inducing ligand (TRAIL, Apo-2L), expressed on activated T cells, is a member of the tumor necrosis factor superfamily. Multiple receptors can selectively induce apoptosis, but most gastric cancer cells are insensitive. In human gastric cancer BGC823 and SGC7901 cells, β-elemene was found to increase the sensitivity of these cells to TRAIL by promoting the formation of death-inducing signaling complex (DISC) in lipid rafts [93]. Tumor-associated macrophages (TAMs), mainly the alterative activated (M2) phenotype, display pro-tumoral property and have become an attractive target for anti-cancer therapy. A study indicated that β-elemene could inhibit the migration, invasion, and epithelial mesenchymal transition of the mouse Lewis lung carcinoma cells promoted by the conditioned medium of M2 RAW264.7 macrophages and regulate the polarization of macrophages from M2 to M1 [94]. Furthermore, radiation (X-ray, 4 Gray) and hypoxia also induced the M2 macrophages’ infiltration and polarization in the mouse Lewis lung carcinoma cells dependent on monocyte chemoattractant protein-1 (MCP-1). β-Elemene was found to have effectively suppressed M2 macrophage recruitment and MCP-1 expression through inhibiting the Prx-1/NF-𝑘B/HIF-1α signaling pathway [95]. Interleukin-23 (IL-23) involves the antigen presentation of dendritic cells (DCs), which are the most potent antigen presenting cells (APCs). Bone marrow-derived dendritic cells (BM-DCs) modified with genes encoding murine IL-23 (DC vaccine) displayed potent clinical applications. It was reported that β-elemene exerted great collaborative anti-tumor effects combined with IL-23-modified DC vaccine via enhancing specific Th1-type and cytotoxic T lymphocyte (CTL) responses against pancreatic carcinoma in the female C_57_BL/6 (B6) mice [96]. 

## 3. Structural Modification of β-Elemene

### 3.1. Reduction and Oxidation Derivatives of β-Elemene 

**Figure 3:**
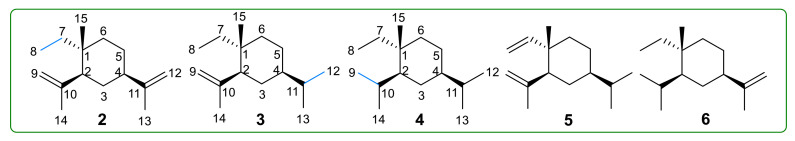
Chemical structures of β-elemene reduction derivatives **2**–**6**.

The structure of β-elemene contains three carbon-carbon double bonds, which are important pharmacophores for its anti-tumor activity. Due to the influence of chemical configuration and steric hindrance, the most active double bond of β-elemene is located at the 7, 8 positions, followed by the 11, 12 double bond, and the 9, 10 double bond was the least active [97]. Compounds **2**–**6** (Figure 3) were five reduction products of β-elemene synthesized via hydrogenation reaction [97,98]. However, since no hydrophilic polar groups are introduced, hydrogenation derivatives did not improve the water solubility of β-elemene, and it is not helpful to improve its physical and chemical properties and biological activity.

**Figure 4:**
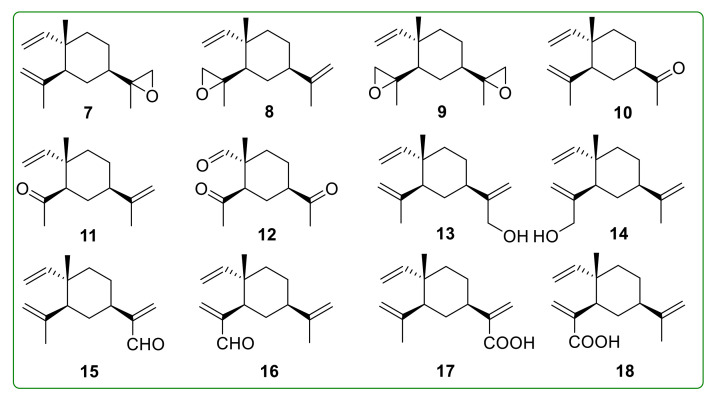
Chemical structures of β-elemene oxidation derivatives **7**–**18**.

Thomas et al. [99] and Maurer et al. [100] employed ozone oxidation to oxidize the double bond of β-elemene into epoxides **7**–**9** and ketones **10**–**12** (Figure 4). However, there is no report on the anti-tumor activity of related oxidation products. Because the double bonds are key anti-tumor pharmacophores, and one or two double bonds of the oxidation product **7**–**12** are destroyed, we speculate that the anti-tumor activity of these oxidation derivatives may not be effectively improved. Oxidation of the substructures other than double bonds can introduce hydrogen bond acceptors and polar groups without changing the pharmacophores, which will enhance the biological activity of the products. Based on this, Bai et al. [101] synthesized 13-β-elemenol (**13**), 14-β-elemenol (**14**), 13-β-elemenal (**15**), 14-β-elemenal (**16**), and the acid derivatives **17** and **18** (Figure 4). Compound **15** and compound **16** exhibited the most potent anti-proliferative effect on A549, HepG-2, and U87 cancer cell lines, with IC_50_ values ranging from 11.61 to 59.55 μM.

**Figure 5:**
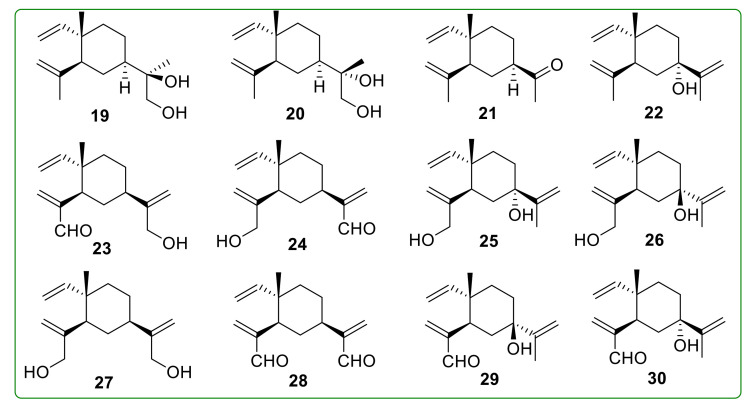
Chemical structures of β-elemene oxidation derivatives **19**–**30**.

Li et al. [102] designed and synthesized several β-elemene hydroxylated derivatives and found only compounds **19**–**21** (Figure 5) displayed comparable anti-proliferation activity with β-elemene against malignant brain tumor cells A172, CCF-STTG1, and U-87MG. Compounds **19** and **20** exhibited the same anti-tumor effect, while compound **21** was inferior to β-elemene. Xie et al. [103] designed and synthesized six β-elemene derivatives **22**–**27** (Figure 5) by SeO_2_-mediated oxidation for the first time. These derivatives showed more potent anti-tumor activity than β-elemene on several tumor cells. Compounds **28**–**30** (Figure 5) were another three β-elemene oxidation derivatives synthesized via a pyridinium dichromate (PDC)-mediated oxidation reaction. Among these compounds, compound **28** exerted significant anti-proliferation activity against A549 and U-87 cells, with IC_50_ values of 9.34 and 2.83 μM, respectively. 

### 3.2. Halogenated Derivatives of β-Elemene

As mentioned above, although the breaking of the unsaturated double bond improved the drug-like properties of β-elemene, this kind of modification negatively affects its anti-tumor activity. Subsequently, under the premise of retaining the double bond, the researchers focused on the optimization of the 13 or 14 methyl groups. The structural modifications of 13 and 14 methyl groups are inseparable from a series of key intermediates, which is the halogenated products of elemene, such as chlorinated β-elemene and brominated products.

**Figure 6:**
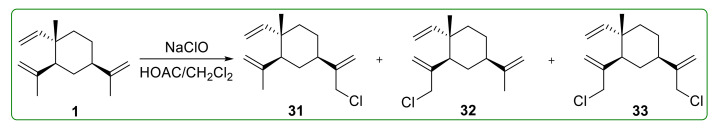
The chlorination reaction and the chemical structures of related products **31**–**33**.

In the chlorination reaction of β-elemene, the chlorination products are mainly 13-chloro-β-elemene (**31**), and a small amount of 14-chloro-β-elemene (**32**) and dichlorination 13,14-bischloro-β-elemene (**33**) products will also be generated (Figure 6). However, the polarity of the 13-chloro-β-elemene and 14-chloro-β-elemene are too similar to be separated. Instead, the mixture of monochloride products is always used as a raw material to prepare the next-step products. Finally, 13-substituted derivatives with better purity can be separated by the polar difference of the subsequent reaction products. Unfortunately, 14-substituted derivatives are always discarded as impurities due to their low content. 

**Figure 7:**
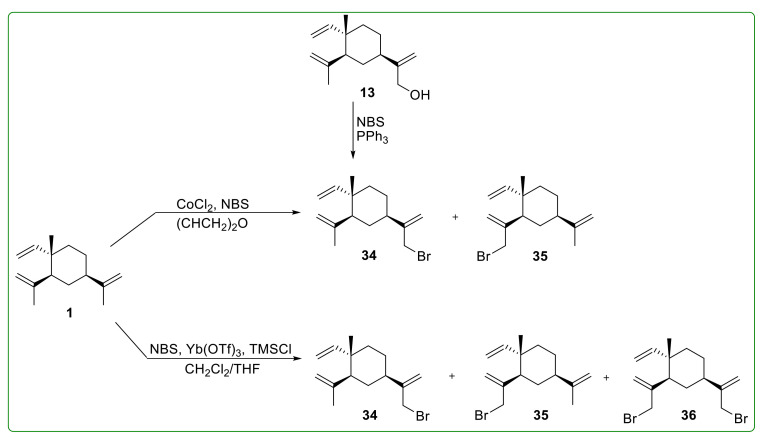
The bromination reaction and the chemical structures of related products **34**–**36**.

Xie et al. [104] successfully separated compound **31** by cyclic preparative liquid chromatography, but compound **32** was still not obtained. Moreover, Chu et al. [104] synthesized 13-bromo-β-elemene (**34**) (Figure 7) and 14-bromo-β-elemene (**35**) (Figure 7) in NBS and transition metal chloride (CoCl_2_) system and these derivatives were directly used for the next reaction without separating. Xie et al. [104] prepared **34**, **35,** and 13, 14-bisbromo-β-elemene (**36**) by using the Yb(OTf)_3_/TMSCl/NBS system. Chen et al. [105] prepared 13-bromo-β-elemene (**34**) with high purity using a slightly more complicated method (Figure 7). They firstly synthesized the mixture of compounds **31** and **32** by the NaClO/CH_3_CO_2_H system and the mixture was directly applied in the next reaction. The above mixture was converted to esters by displacement of chloride with CH_3_CO_2_Na, then to a mixture of alcohols (**13** and **14**) via the hydrolysis under alkaline conditions. Finally, **13** was obtained after high-performance liquid chromatography (HPLC) purification, which was further converted to **34** using the NBS/PPh_3_ system.

**Figure 8:**
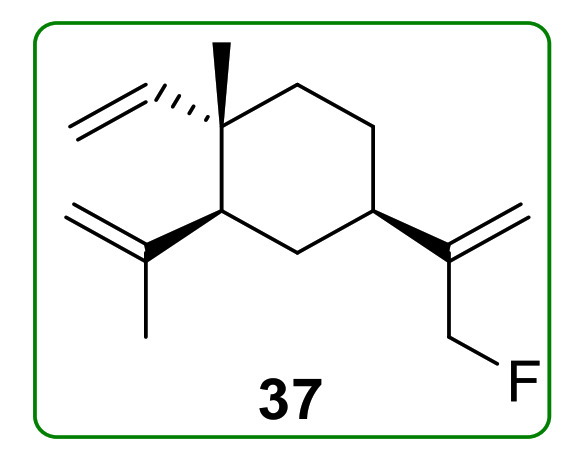
Chemical structures of β-elemene fluorinated derivatives **37**.

13-Fluoro-β-elemene (**37**) (Figure 8) was a fluorinated compound of β-elemene and displayed an anti-tumor effect comparable to that of β-elemene on NSCLC cells. Furthermore, the MTT assay indicated that the IC_50_ values against H460 cells of β-elemene and compound **37** were 70.6 and 62.5 μg/mL, respectively [73].

### 3.3. Amine Derivatives of Β-Elemene

**Figure 9:**
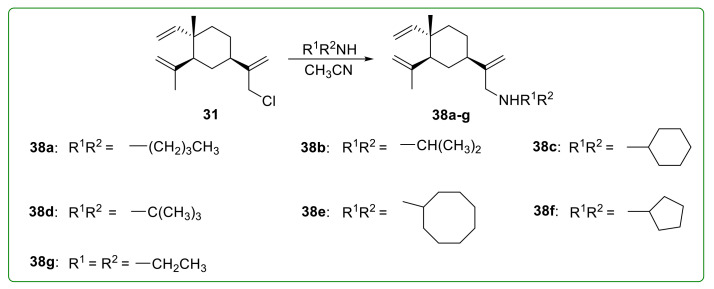
Chemical structures of β-elemene aminated derivatives **38a**–**g**.

Sun et al. [106] firstly synthesized 13-monosubstituted amines of β-elemene derivatives **38a**–**g** (Figure 9) by strictly controlling reaction conditions. Among them, compounds **38b**, **38c,** and **38f** showed potent anti-tumor activity on Hela cell lines with IC_50_ values of 0.04, 3.39, and 0.52 μM, respectively, which were superior to that of β-elemene. Further studies demonstrated that the anti-tumor activity of these derivatives was associated with blocking the cell cycle at the G_1_ phase by reducing Rb phosphorylation and cyclin D_1_ protein expression.

**Figure 10:**
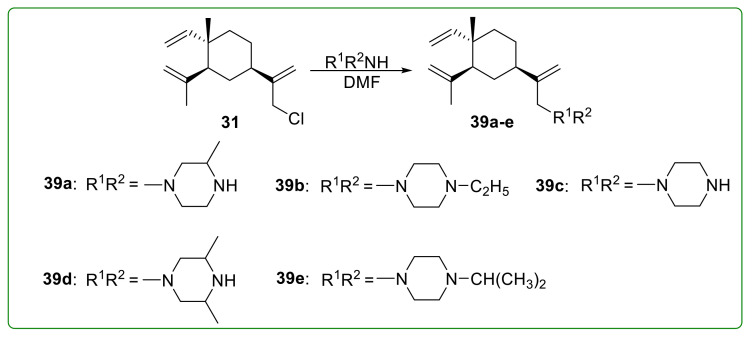
Chemical structures of β-elemene aminated derivatives **39a**–**e**.

Yu et al. [107] designed and synthesized five novel β-elemene piperazine derivatives **39a**–**e** (Figure 10) to improve its anti-tumor activity. The inhibitory effect of these derivatives on cell growth was more potent than β-elemene, and their IC_50_ values were less than 15 μM. These derivatives could also activate death receptor-mediated and mitochondrial-mediated apoptosis pathways *via* increasing ROS production and decreasing the cellular FLICE-inhibitory protein (c-FLIP) level.

**Figure 11:**
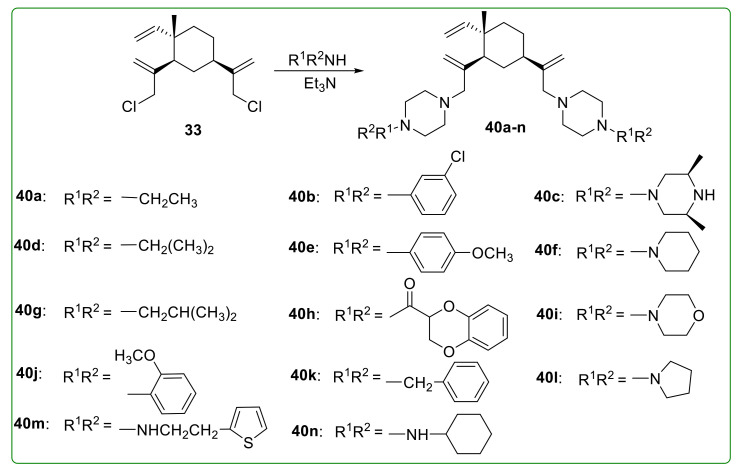
Chemical structures of β-elemene aminated derivatives **40a**–**n**.

Xu et al. [108] designed and synthesized fourteen 13, 14-disubstituted derivatives **40a**–**n** (Figure 11) of β-elemene by introducing hydrophilic moiety. The water solubility and anti-tumor activity of obtained products were successfully increased. Compounds **40c**, **40m**, and **40n** revealed potent anti-tumor activity in vitro by decreasing the mTOR or PKB level, with IC_50_ values less than 5 μM on K562 cells. Further investigation indicated that compound **40c** also displayed potent anti-tumor activity against several other cell lines, including gastric, breast, lung, liver, ovarian, colon cancer, and leukemia cells, with an average IC_50_ value of 3.44 μmol/L [109].

In short, the introduction of amine-derived pharmacophores into the β-elemene structure can not only improve their physical and chemical properties but also greatly enhance their anti-tumor activity in vitro. Therefore, this structural modification method is a practical strategy.

### 3.4. Ether Derivatives of β-Elemene

**Figure 12:**
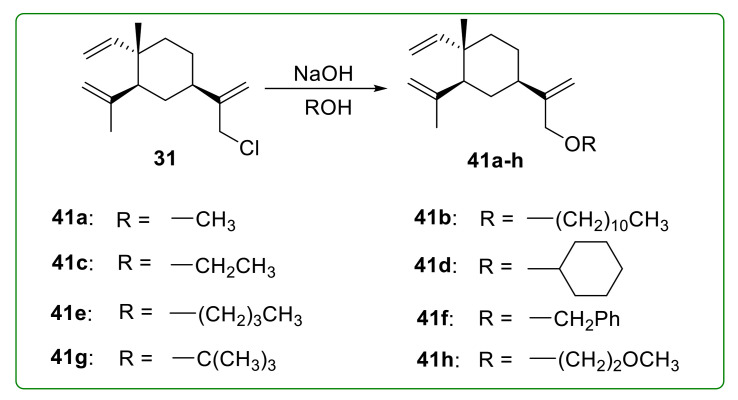
Chemical structures of β-elemene ether derivatives **41a**–**h**.

Compounds **41a**–**h** (Figure 12) were eight etherified β-elemene derivatives with different aliphatic or aromatic substituents. However, the anti-proliferative activity of these derivatives was not effectively improved. Only compound **41c** showed slightly more potent anti-proliferative activity than β-elemene, with an IC_50_ value of 105.9 μM [106]. These results indicated that etherification modification of β-elemene was not a successful strategy to obtain much more potent β-elemene derivatives. 

**Figure 13:**
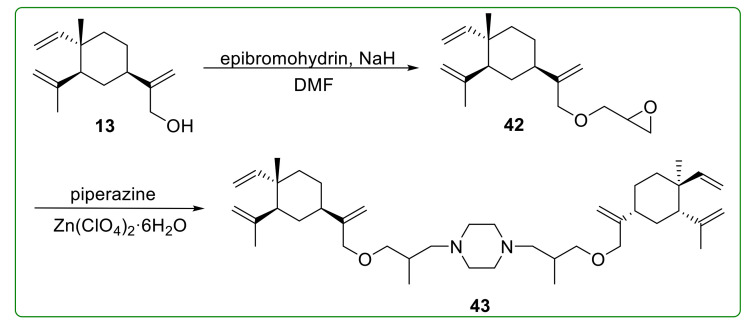
Chemical structures of β-elemene ether derivative **43**.

Ether **43** (Figure 13) was a novel β-elemene dimer derivative with a piperazine moiety, which displayed remarked anti-proliferative effect with an IC_50_ value less than 11 μM. More importantly, the combination of compound **43** and cisplatin could effectively reverse the drug resistance of A549/DPP cells. Further studies indicated that compound **43** could arrest the cell cycle at the G_2_ phase and induce cell death in a mitochondrial-dependent apoptosis way [110].

### 3.5. Ester Derivatives of β-Elemene

**Figure 14:**
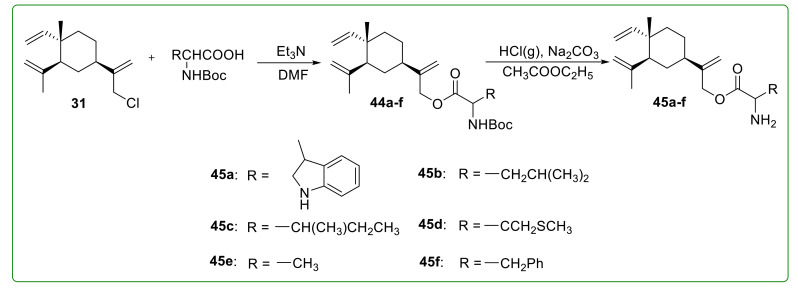
Chemical structures of β-elemene ester derivatives **45a**–**f.**

**Figure 15:**
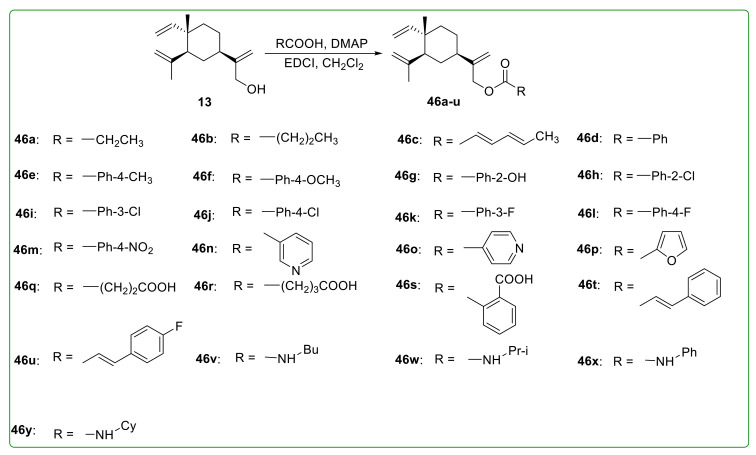
Chemical structures of β-elemene ester derivatives **46a**–**y**.

As illustrated in Figure 14, Zhang et al. [111] designed and synthesized six esterified derivatives of β-elemene (**45a**–**f**) to improve the water solubility. These derivatives exhibited significant anti-tumor activity compared to β-elemene against Hela, HL-60, and SGC-7901 cells. Chen et al. [112] prepared a series of 13-β-elemene ester derivatives **46a**–**u** (Figure 15) to improve the activity and half-life of β-elemene and investigated the antioxidant activity in human umbilical vein endothelial cells (HUVECs). The results proved that only compound **46g** showed better antioxidant activity than β-elemene. Liu et al. [113] synthesized five different β-elemene ester and carbamate derivatives (**46v**–**y**) (Figure 15). However, the anti-tumor activity was not significantly improved.

**Figure 16:**
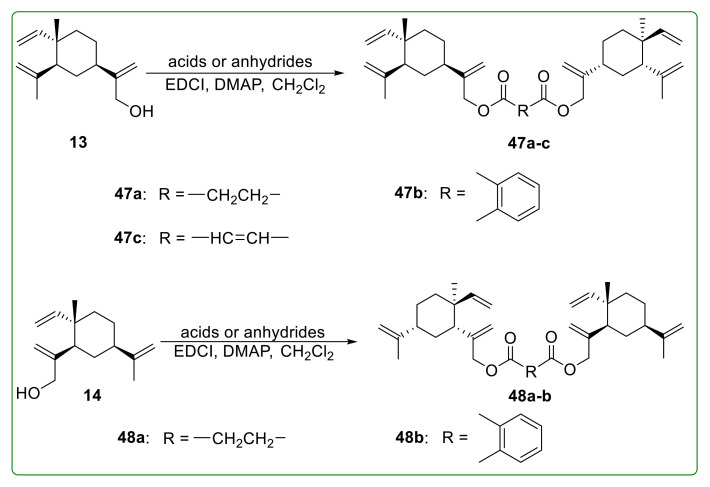
Chemical structures of β-elemene ester derivatives **47a**–**c** and **48a**–**b**.

Chen et al. [114] also designed and synthesized several β-elemene dimer ester derivatives and investigated their antioxidant activity on HUVECs. Furthermore, 13-monosubstituted dimers **47a** and **47b** (Figure 16) demonstrated the most effective antioxidant activity associated with the production inhibition of ROS, which were better than the positive control vitamin E. Intriguingly, the antioxidant activity of 14-monosubstituted dimers **48a** and **48b** (Figure 16) was weaker than 13-monosubstituted dimers **47a** and **47b**. Moreover, compound **47c** (Figure 16) showed no obvious cytotoxicity to HUVECs and displayed more active antioxidant activity than vitamin E and compound **47a**. Further studies indicated that compound **47c** could increase the expression of superoxide dismutase and nitric oxide release in cells, and significantly reduce intracellular malondialdehyde and lactate dehydrogenase [105]. 

### 3.6. Amino Acid Derivatives of β-Elemene

**Figure 17:**
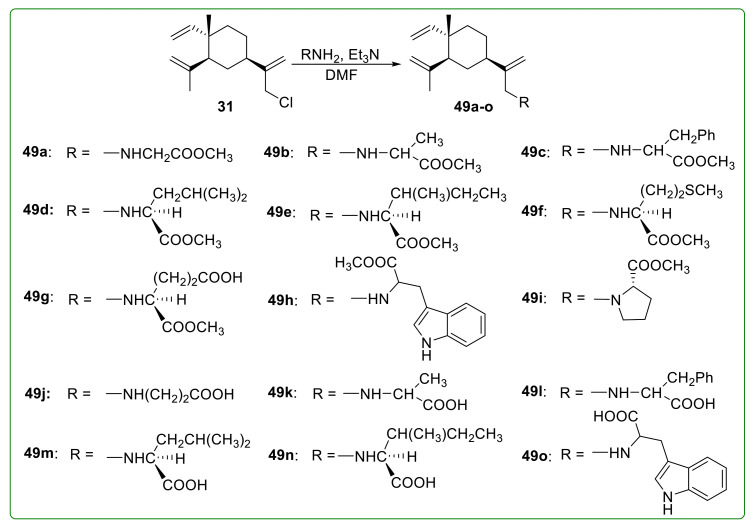
Chemical structures of β-elemene amino acid derivatives **49a**–**o**.

It is a feasible modification strategy to design β-elemene amino acid derivatives to enhance the anti-tumor effect by selectively acting on the amino acid transport system of tumor cells. The corresponding derivatives could also possess better drug-like properties, such as higher solubility and more hydrogen donors or acceptors. Therefore, compounds **49a**–**o** (Figure 17) were designed and prepared following this idea. All the β-elemene amino acid ester derivatives showed potent anti-tumor activity superior to β-elemene on HeLa, SGC-7901, and HL-60 cells. Compound **49h** displayed the best anti-proliferative activity with IC_50_ values of 27.5 μM on SGC-7901 cells, 14.6 μM on HeLa, and 25.4 μM on HL-60 cells, respectively [115]. The deeper investigation indicated that compound **49h** could arrest the cell cycle at the G_2_/M phase and induce apoptosis in hepatocellular carcinoma (HCC) cells [116].

### 3.7. Glycoside Derivatives of β-Elemene 

**Figure 18:**
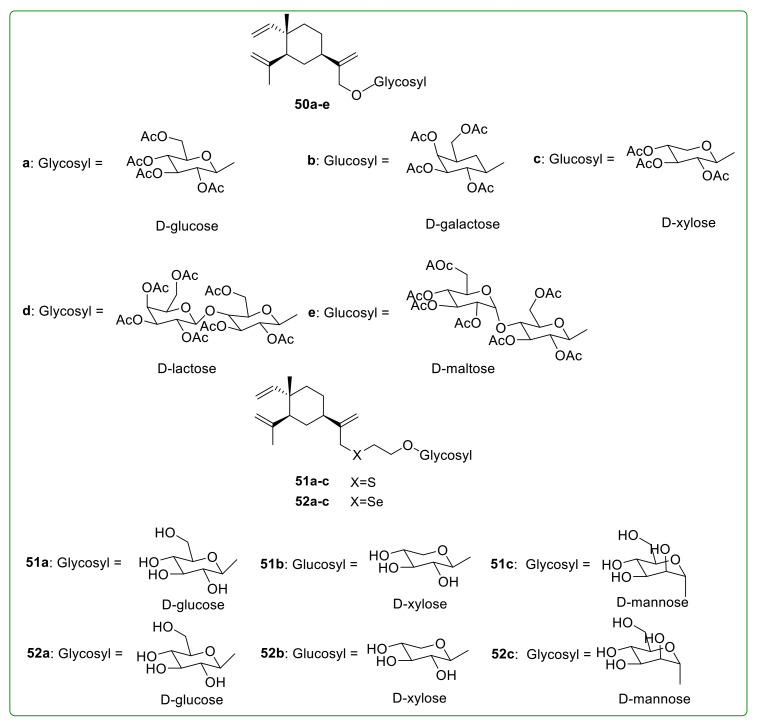
Chemical structures of β-elemene glycosylated derivatives **50**–**52**.

Carbohydrates are a type of typical hydrophilic substance widely existing in nature, playing a vital and complex role in many physiological activities, such as cell recognition and signal transduction [117]. Yang et al. [118] synthesized five β-elemene glycosylation derivatives **50a**–**e** (Figure 18) via introducing glycoside scaffolds into β-elemene to increase the water solubility and anti-tumor activity. Subsequently, Yang et al. [119] synthesized six β-elemene glycosylation derivatives **51a**–**c** and **52a**–**c** (Figure 18) containing sulfur (S) and selenium (Se) atoms. However, the anti-tumor activity of these derivatives has not been reported. In theory, this is a targeted optimization strategy based on the high lipophilic nature of β-elemene, but whether it is feasible still needs to be verified by further biological evaluations.

### 3.8. Radioactive Derivatives of β-Elemene

**Figure 19:**
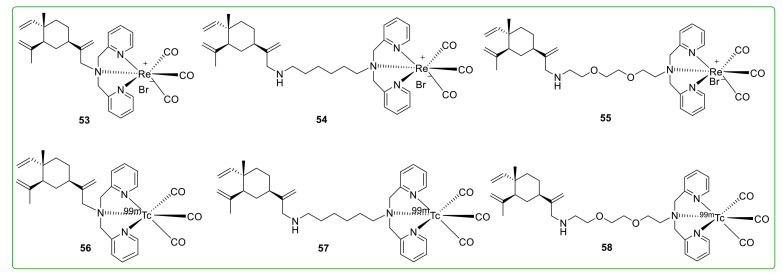
Chemical structures of radioactive derivatives **53**–**58**.

Sun et al. [106] synthesized three β-elemene rhenium derivatives of **53**–**55** (Figure 19). These derivatives exhibited anti-proliferative activity similar to β-elemene by arresting the cell cycle at the G_1_ phase through reducing Rb phosphorylation and cyclin D_1_ protein expression in HeLa cells. Sun et al. [120] then continued to prepare three ^99m^Tc(CO)_3_-β-elemene derivatives **56**–**58** (Figure 19) with more favorable water solubility. In terms of the oil–water partition coefficient, compound **58** was about twenty times lower than β-elemene. 

### 3.9. NO-Donating Derivatives of β-Elemene

**Figure 20:**
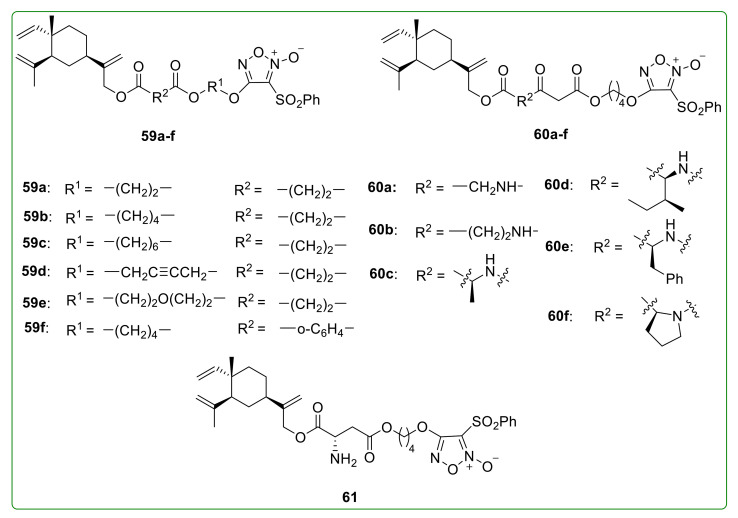
Chemical structures of β-elemene NO-donating derivatives **59**–**61**.

Nitric oxide (NO) is an important signaling molecule with various physiological functions [121]. High levels of NO play a vital role in inducing cell apoptosis, inhibiting tumor metastasis, and sensitizing drug-resistant tumor cells to chemotherapy and radiotherapy [122]. Furoxan is a classic type of NO donor, which produces high levels of NO in vitro and inhibits the growth of tumors in vivo [123]. Chen et al. [124] designed and synthesized a series of β-elemene NO-donating derivatives **59**–**61** (Figure 20) by introducing the furoxan NO donor group. Among these compounds, **59a** displayed promising anti-tumor activity by arresting the cell cycle at the G_2_ phase and inducing cell apoptosis on U-87 and SGC-7901 cells. Furthermore, treatment with compound **59a** (60 mg/kg, i.v.) once a day after three weeks showed a cancer inhibitory ratio (TIR) of 64.8% compared to β-elemene (49.6%) on a H22 liver cancer xenograft mouse model.

## 4. Conclusions

As a second-line anti-tumor drug that has been clinically used for more than 20 years in China, natural product β-elemene exerts clinical anti-tumor efficacy through various mechanisms of action, with promising clinical and research significance. In recent years, clinical trials have further confirmed that β-elemene can also be used as a sensitizer and synergist of chemotherapeutic drugs, and can effectively reduce the side effects and adverse reactions of traditional chemotherapeutic drugs, which has successfully further expanded its clinical application. However, in terms of the structure of β-elemene itself, its excessive polarity lipophilic physicochemical profile also leads to a poor drug-like property. Therefore, structural modifications and optimizations are promising strategies to obtain better candidates. Although halogenation, etherification, esterification, amination, and other modifications have been performed, the relative research is still few and not deep enough. All in all, as a rare anti-tumor natural lead compound, β-elemene deserves more in-depth and effective structural modification to discover novel β-elemene derivatives with improved drug-like properties and potent anti-tumor activity in vivo.

## Figures and Tables

**Table 1:** The specific information of elemene oral emulsion and elemene injection.

	Elemene Oral Emulsion	Elemene Injection	
Ingredient list	85% β-elemene and 15% γ- and δ-elemene	85% β-elemene and 15% γ- and δ-elemene
**Description**	opalescent homogeneous emulsion	opalescent homogeneous emulsion
**Indication**	for adjuvant treatment of esophageal cancer and gastric cancer to improve symptoms	lung cancer, liver cancer, esophageal cancer, nasopharyngeal cancer, brain cancer, and bone metastasis; intervention, intracavitary chemotherapy, and carcinoma hydrothorax treatment
**Side effects**	digestive tract reactions: nausea, vomiting, diarrhea, and occasionally loss of appetite; decreased hemoglobin; decreased leukocytes	phlebitis; fever; local pain; allergic reaction; mild digestive tract reaction
**Specification**	20 mL: 176 mg	20 mL: 88 mg
**Pharmacokinetics** [5,6]	t_1/2_ = 65 min (i.v.) t_1/2_ = 126 min (i.p.)	bioavailability = 18.8%

## Abbreviations

ABC, ATP-binding cassette; Apaf-1, apoptosis protease-activating factor-1; APCs, antigen presenting cells; Atg4B, autophagy-related gene 4B; BCRP, breast cancer resistance protein; bFGF, basic fibroblast growth factor; Bid, binding interface database; BM-DCs, bone marrow-derived dendritic cells; CDC, cell division cycle; CDK, cyclin-dependent kinases; CFDA, China Food and Drug Administration; c-FLIP, cellular FLICE-inhibitory protein; CTL, cytotoxic T lymphocyte; CTR1, copper transporter 1; DCs, dendritic cells; DISC, death-inducing signaling complex; DNMT, DNA methyltransferase; EGFR, epidermal growth factor receptor; EMT, epithelial-mesenchymal transition; ERCC-1, excision repair cross-complementation group-1; ER, endoplasmic reticulum; EZH2, the enhancer of zeste homolog 2; FADD, fas-associated death domain; FAK, focal adhesion kinase; FoxO 3a, forkhead box class O 3a; 5-FU, 5-fluorouracil; GBM, glioblastoma multiforme; GCSCs, gastric cancer stem-like cells; GMFβ, glia maturation factor β; GSLCs, glioma stem-like cells; HBXIP, hepatitis B X-interacting protein; HCC, hepatocellular carcinoma; HIF-1α, hypoxia inducible factor-1α; HPLC, high-performance liquid chromatography; hTERT, human telomerase reverse transcriptase; HUVE, human umbilical vein endothelial; IAP, inhibitor of apoptosis; IGFBP 1, insulin-like growth factor-binding proteins 1; i.p., intraperitoneal injection; i.v., intravenous injection; lncRNA, long noncoding RNA; MAT3, metastasis-associated protein 3; MCP-1, monocyte chemoattractant protein-1; MDR, multidrug resistance; MMPs, matrix metalloproteinases; Mta-1, metastasis-associated gene 1; mTOR, mammalian target of rapamycin; NO, nitric oxide; NSCLC, non-small-cell lung cancer; PAK1, p21-activated protein kinase 1; PAK1IP1, PAK1-interacting protein; PARP, poly ADP-ribose polymerase; PDC, pyridinium dichromate; PE, phosphatidylethanolamine; P-gp, P-glycoprotein; PKM2, pyruvate kinase M2; Prx-1, peroxiredoxin-1; PTEN, phosphatase and tensin homolog deleted on chromosome ten; PUMA, p53-up-regulated modulator of apoptosis; ROS, reactive oxygen species; Stat3, signal transducer and activator of transcription 3; TAMs, tumor-associated macrophages; tBID, truncated binding interface database; TGF-β, transforming growth factor-β; TOPO I, topoisomerase I; TRAIL, tumor necrosis factor-related apoptosis-inducing ligand; uPA, urokinase-type plasminogen activator; uPAR, uPA receptor; VEGF, vascular endothelial growth factor; XIAP, X-linked inhibitor of apoptosis
